# Fluorescent Zn(II)-Based Metal-Organic Framework: Interaction with Organic Solvents and CO_2_ and Methane Capture

**DOI:** 10.3390/molecules27123845

**Published:** 2022-06-15

**Authors:** Sifani Zavahir, Hamdi Ben Yahia, Julian Schneider, DongSuk Han, Igor Krupa, Tausif Altamash, Mert Atilhan, Abdulkarem Amhamed, Peter Kasak

**Affiliations:** 1 Center for Advanced Materials, Qatar University, Doha P.O. Box 2713, Qatar; fathima.z@qu.edu.qa (S.Z.); dhan@qu.edu.qa (D.H.); igor.krupa@qu.edu.qa (I.K.); 2 Qatar Environment & Energy Research Institute, Hamad Bin Khalifa University, Doha 34110, Qatar; benyahia_hamdi@yahoo.fr (H.B.Y.); taltamash@hbku.edu.qa (T.A.); 3 Department of Materials Science and Engineering, and Center for Functional Photonics (CFP), City University of Hong Kong, 83 Tat Chee Avenue, Hong Kong 999077, China; julianschneider86@gmx.de; 4 Department of Chemical and Paper Engineering, Western Michigan University, Kalamazoo, MI 49008, USA; mert.atilhan@wmich.edu

**Keywords:** MOF, citric acid derivative, TPDCA

## Abstract

Adsorption of carbon dioxide (CO_2_), as well as many other kinds of small molecules, is of importance for industrial and sensing applications. Metal-organic framework (MOF)-based adsorbents are spotlighted for such applications. An essential for MOF adsorbent application is a simple and easy fabrication process, preferably from a cheap, sustainable, and environmentally friendly ligand. Herein, we fabricated a novel structural, thermally stable MOF with fluorescence properties, namely Zn [5-oxo-2,3-dihydro-5H-[1,3]-thiazolo [3,2-a]pyridine-3,7-dicarboxylic acid (TPDCA)] • dimethylformamide (DMF) •0.25 H_2_O (coded as QUF-001 MOF), in solvothermal conditions by using zinc nitrate as a source of metal ion and TPDCA as a ligand easy accessible from citric acid and cysteine. Single crystal X-ray diffraction analysis and microscopic examination revealed the two-dimensional character of the formed MOF. Upon treatment of QUF-001 with organic solvents (such as methanol, isopropanol, chloroform, dimethylformamide, tetrahydrofuran, hexane), interactions were observed and changes in fluorescence maxima as well as in the powder diffraction patterns were noticed, indicating the inclusion and intercalation of the solvents into the interlamellar space of the crystal structure of QUF-001. Furthermore, CO_2_ and CH_4_ molecule sorption properties for QUF-001 reached up to 1.6 mmol/g and 8.1 mmol/g, respectively, at 298 K and a pressure of 50 bars.

## 1. Introduction

Carbon dioxide sequestration technology is in line with recommendations of the IPCC Special Report, which set a goal for the reduction of global carbon dioxide emissions by about 45% by 2030 and achievement of net-zero by 2050 compared to 2010 levels [1,2]. Additionally, as greenhouse gases have a direct impact on human health and the environment, safe separation of hazardous gases such as methane and carbon dioxide is essential but technically challenging [3,4,5,6]. In order to strengthen the renewable gas separation technology, there have been several attempts focused on the use of various biomolecule-based (citric, malic, and lactic acid) materials [7,8,9]. Similarly, Zn ion-based metal-organic frameworks (MOFs) were gaining interest in gas capturing and separation applications [10,11,12].

In general, MOFs have drawn keen research interest as a family of crystalline materials; they consist of inorganic metal ions connected with organic ligands such as carboxylate, phosphonate or heteroaromatics and others [13,14]. The coordination ability of the ligands is the main factor that determines the assembly of MOFs into one-, two-, or three-dimensional architectures that subsequently dictate their properties and possible application [15,16,17]. Due to tunable architecture, high surface area, and porosity [18], MOFs have already found vast utilization in catalysis [19], gas storage [20] and separation [21], (bio)imaging, drug delivery, optoelectronics, and sensing [22,23]. One of the important classes of MOFs is the luminescent type, where fluorescence or phosphorescence results from the absorption of light at a radiative excitation state that leads to photon emission. Inherent porosity and a precise crystal structure are advantages that determine the selectivity of interaction for sensing applications of luminescent MOFs [24], which can be used for sensing explosives, ions, biomolecules, toxic and volatile organic compounds, temperature, pH, etc. [25,26]. Luminescence can originate from the organic linker and metal node [27] as the luminescent part or by inserting luminescent guest molecules [28]. One of the most commonly studied origins of luminescence in MOFs is emission from the organic linker, where the ligand molecule acts as a rigid structural component and simultaneously provides the emissive character of the structure. Thus, such ligands as a building moiety are one of the most crucial components of luminescent MOFs, and there is high demand for cheap, sustainable, and environmentally benign ligands with tailored properties.

Citric acid is a natural, sustainable, cheap, and functional low-molecular-weight carbon source [29]. Recently, there has been extensive research interest in citric acid-based functional materials [30,31,32], such as citric-based carbon dots; this molecule was also identified as a source of a low-molecular-weight fluorophore formed under certain synthetic conditions [33,34,35,36], as well as major luminescent molecular fluorophore [37,38,39]. This is because citric acid can form stable fluorophore derivatives by reaction with amines [40], α,β-diamines, α-amino acids, and α,β-heteroatom amines [41]. Thus, dicarboxylic acid derivatives as potential ligands for MOFs can be prepared in reaction with some multifunctional natural amino acids. In the reaction of citric acid with natural amino acid L-cysteine, 5-oxo-2,3-dihydro-5H-[1,3]-thiazolo [3,2-a]pyridine-3,7-dicarboxylic acid (TPDCA) [42,43] has been synthesized and further applied as a component of fluorescent biodegradable polymers [44,45] and other soft materials [46,47,48,49]. TPDCA also has been identified as a key component for induction of gelation of natural polysaccharide-alginate [50] and related carbon dots [51,52]. In this work, TPDCA was employed as a ligand for the formation of different MOF structures. The crystal structure was confirmed for Zn-based structure as QUF-001, and used for solvent interaction investigation. Moreover, carbon dioxide and methane gases have been successfully tested to quantify the adsorption–desorption range in prepared Zn-based QUF-001 structures at high pressures and temperatures.

## 2. Results and Discussion

Zn-, Cu- and Fe-based MOFs were synthesized using the TPDCA ligand, and the obtained samples were coded as QUF-001, QUF-002 and QUF-003, respectively (Figure S1). The three MOF samples were prepared using DMF as a solvent as well as a base precursor for carboxylate formation in solvothermal conditions. The three central elements were chosen on the basis of their respective importance in the human body, as they can lead to essential bio-inorganic materials [53,54,55]. These different central metal nodes were expected to result in slightly different geometry and open channels in the host crystal structure.

**Figure 1:**
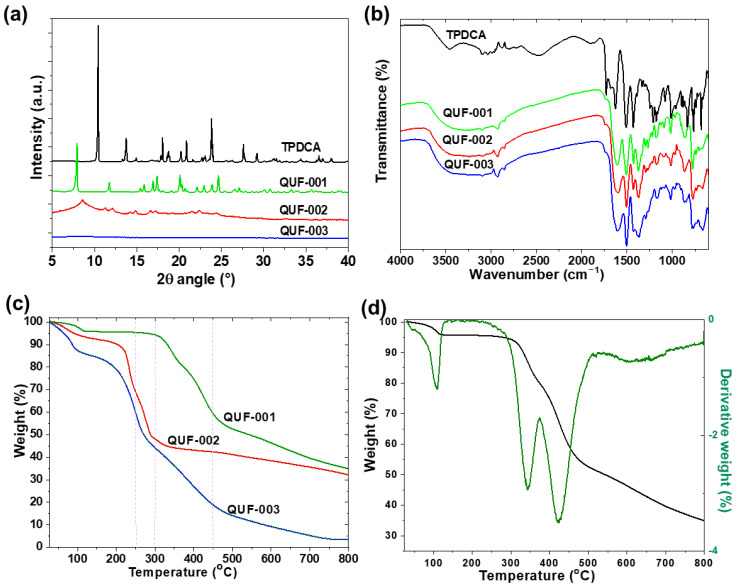
(**a**) XRPD patterns and (**b**) FTIR spectra of TPDCA ligand, QUF-001, QUF-002 and QUF-003. (**c**) TGA curves of QUF-001, QUF-002 and QUF-003. (**d**) TGA and DTA curves of QUF-001.

X-ray powder diffraction (XRPD) patterns of the prepared samples are depicted in Figure 1a. Only the Zn-MOF (QUF-001) was well-crystallized with sharp diffraction peaks. Therefore, a full pattern matching was performed on this MOF only, using the JANA 2006 program, which confirmed the purity of this compound as depicted in Figure S2. Furthermore, the refined cell parameters were in good agreement with those obtained from single crystal XRD data (Table 1). The Cu-MOF (QUF-002), although formed, was not as well-crystallized as the Zn-based one, as its XRPD pattern (Figure 1a) showed significant peak broadening. The crystal structure of Cu-MOF could not be solved from XRPD, and all attempts to grow single crystals failed. No diffraction peaks were observed for Fe-based QUF-003, which indicated that the sample was amorphous and the Fe-MOF was not formed. This information is in line with the visual observation where the solid powder of QUF-003 appeared to be a precipitate rather than a polycrystalline substance.

FTIR spectra of the three MOF samples had similar peaks; they were partly different from that of the TPDCA ligand pattern as depicted in Figure 1b. TPDCA showed several FTIR bands not present in the MOF structures, centered at 3450, 2500, and 1724 cm^−1^, which could be assigned to O-H stretching, intermolecular bonding from O-H stretching, and C=O stretching of carboxylic acid groups, respectively. The absence of such bands in the MOF structures was due to transformation of the carboxylic acid group of TPDCA to carboxylate during slow formation of the base from DMF decomposition, as well as due to formation of multidentate ligand making coordination bonds with the metal central node. FTIR absorption signals at 3042, 1630 1517, 1430, 1070 and 680 cm^−1^ in TPDCA were attributed to aromatic C-H stretching, amidic C=O stretching, C-N stretching, C-O stretching, C-N bending and S-C stretching modes [56], respectively. The amidic C=O stretching vibration overlapped with the carboxylate stretching vibration at 1607, 1603 and 1604 cm^−1^ for Zn-, Cu- and Fe-based structures [56], respectively. Moreover, a broad peak related to hydrogen bonding was centered at 3300 cm^−1^. On the other hand, the ligand TPDCA differed in absorption peaks for hydrogen bonding at 3460 cm^−1^ and an additional absorption peak belonging to the carboxylic acid functionality at 1732 cm^−1^. This observation assumed that the coordination of the TPDCA ligand to the central metal ion in the three MOF structures was due to similar carboxylate functionalities.

The thermogravimetric analysis (TGA) data for the three samples are given in Figure 1c. The three samples exhibited significantly different thermal behaviors, which was expected since their XRPD patterns were significantly different. QUF-003 was the least heat-stable sample, with ~15%, ~55% and ~90% weight losses at 150 °C, 300 °C, and 800 °C, respectively. This indicated a large water or solvent inclusion that was related to loss at 150 °C and further organic residue degradation at relatively low temperature until 300 °C. Additionally, stable residue of only about 10% after heating to 800 °C indicated low abundance of metallic-inorganic components in sample. Sample QUF-002 contained 6% moisture and volatile compounds, and further decomposition started at 200 °C, reaching 50% of initial mass. Residual mass of 67% after heating to 800 °C indicated Cu as the metallic component in MOF, whereas QUF-001 was the most stable sample, with only ~4%, ~45% and ~65% of weight lost at 300 °C, 500 °C, and 800 °C, respectively. The first-order derivative curve of the TGA of QUF-001 is given in Figure 1d, and shows three weight loss points. The first one at 110 °C with an initial weight loss of 4% can be attributed to the removal of moisture/trapped H_2_O and DMF molecules from the channels and pores of this MOF. Two distinctly separated peaks at 330 °C and 420 °C accounting for ~45% weight loss remained during the thermal decomposition of the sample, and it could be assumed that led to the collapse of the crystal structure.

Thus, the analysis based on the techniques discussed above suggests that out of the three samples prepared in this study, only QUF-001 based on Zn exhibited high crystallinity and good thermal stability. Hence, QUF-001 was further employed to evaluate the solvent inclusion properties and gas storage capacity of the MOF based on the TPDCA ligand.

**Figure 2:**
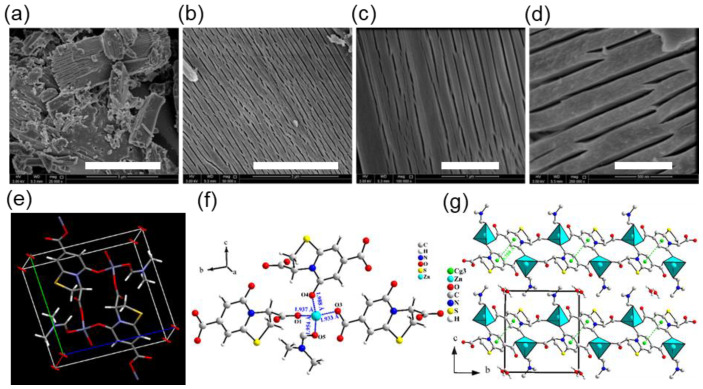
SEM images of QUF-001 at different magnifications, namely (**a**) 25,000×, (**b**) 50,000×, (**c**) 100,000× and (**d**) 200,000×, with scale bars indicating 5 microns, 3 microns, 1 micron and 500 nm, respectively. (**e**) Scheme showing the Zn(II) coordination in the QUF-001 unit cell and (**f**) the coordination sphere of the zinc cation. (**g**) View of the layered structure of QUF-001 along the *a*-axis. The green dashed lines correspond to π–π interactions between the six-membered rings forming TPDCA linkers. *Cg*3 is the centroid of the six-membered ring.

SEM images of QUF-001 demonstrate a well-ordered lamellar-like structure as visualized in Figure 2a–d. The material possesses a two-dimensional character and lamellar structure with low magnitude cracks between them, indicating lower interaction in interlamellar spacing. On the other hand, the polycrystalline form and X-ray single-crystal refinement measurements performed on QUF-001 revealed that the compound C_12_H_12_N_2_O_6_SZn•0.25H_2_O crystallizes in the triclinic crystal system with the space group *P*-1 (Table 1 and Figure 2e). Most of the atomic positions were found by the direct method using SIR2004 [57]. With isotropic atomic displacement parameters (ADPs), the residual factors converged to the value *R*(*F*) = 0.0987 and *wR*(*F*^2^) = 0.2061 for 89 refined parameters and 1762 observed reflections. At this stage of the refinement, the chemical formula C_12_N_2_O_6_SZn could not be equilibrated yet. After adding H atoms and applying restrictions on their positions and (ADPs), the chemical formula became C_12_H_12_N_2_O_6_SZn, and the residual factors decreased only slightly to *R*(*F*) = 0.0956 and *wR*(*F*^2^) = 0.1971. By refining the anisotropic ADPs of all atoms except the H atoms, the residual factors converged to the value *R*(*F*) = 0.0473, *wR*(*F*^2^) = 0.1103 and S= 1.59 for 199 refined parameters. The Fourier difference showed a very weak electron density residue along the MOF tunnels. Therefore, a water molecule was included in the crystal structure. The refinement of the occupancy of oxygen from the water molecule showed a significant decrease from 1 to 0.25. Consequently the occupancies of the water molecule were restricted to 0.25, leading to the chemical formula C_12_H_12_N_2_O_6_SZn•0.25H_2_O, for which the final residual factors were for *R*(*F*), *wR*(*F*^2^) and S values 0.042, 0.106, 1.09, respectively, as provided in Table 1 and Tables S1. Further crystallographic data for the atomic positions and anisotropic ADPs are given in Tables S2 and S3, respectively. Further details on the structural refinement may be obtained from the Cambridge Crystallographic Data Centre (CCDC), by quoting the Registry No. CCDC 2120295 [58]. 

Since a structural disorder of the water molecules was observed along the MOF tunnels, it could indicate that the *P*-1 symmetry was higher than the true symmetry. Therefore, a second refinement was conducted using the space group *P*1. The atomic positions from the first refinement were used as a starting model. By reducing the symmetry from *P*-1 to *P*1, we doubled the number of atoms. The refinement led to residual factors very similar to those from the first refinement; however, most of the atoms displayed a non-positive, definite ADP matrix. Consequently, the MOF structure was considered to be centrosymmetric (space group *P*-1), where each carboxylate group formed from carboxylic acid by decomposition of DMF binds to one Zn(II) in a monodentate fashion. Zn(II) ions have a tetrahedral coordination geometry with two oxygen atoms from a particular carboxylate from TPDCA molecules and two coordination complexes from amidic oxygen atoms from TPDCA and dimethylformamide molecules, as shown in Figure 2b. Each zinc atom interconnects three TPDCA molecules at different positions and one DMF molecule (Figure 2f), forming a 2D framework in the (001) plane (Figure 2g). The Zn-O distances for O1, O3, O4 and O5 are 1.937(3) 1.933(3), 1.987(3) and 1.955(3) Å, respectively, and the angles between carbon, oxygen and zinc atoms for C1-O1-Zn1, C7-O3-Zn1, C9-O4-Zn1 and C10-O5-Zn1 are 118.8(3), 118.3(3), 132.0(2) and 121.5(3)°, respectively. A packing diagram of QUF-001, viewed down the *a*-axis, is given in Figure 2g. Coordination in crystallographic unit consists of two 14-atom rings, and a two-dimensional structure with Zn(II) centered coordination is due to a peripheral DMF molecule oriented to coordination lamellar structure, as shown in Figure 2d. It is worth noting that no hydrogen bonds connecting the different layers were observed. Even the offset π–π interactions between TPDCA molecules [*Cg*3-*Cg*3i = 3.758(2) Å, interplanar distance = 3.3618 (16) Å, slippage = 1.679 Å, α = 0°, *Cg*3 was the centroid of the six-membered ring, symmetry code (i): 1-X,-Y,1-Z] existed only within a single layer and not between layers, which confirms that the structure is bi-dimensional in the (001) plane (see the green dashed lines in Figure 2d).

**Figure 3:**
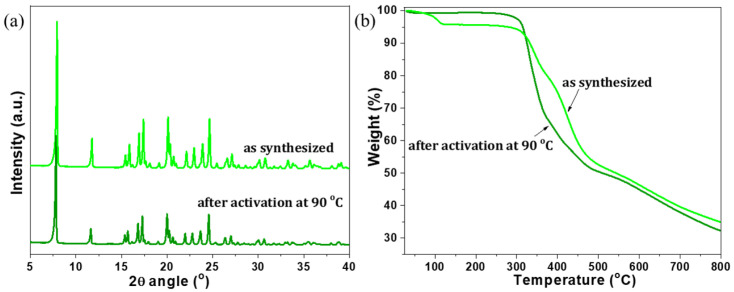
(**a**) XRPD patterns and (**b**) TGA curves of QUF-001, as synthesized and after activation at 90 °C for 16 h.

After confirming the crystallographic structure of Zn-MOF, QUF-001 was activated to remove possible intercalated solvent molecules. During the MOF fabrication, the solvent trapping into pores was rather obvious. Thus, activation is important to have complete accessibility of the pores and to obtain the guest free pores. Thermal activation is a simple and effective method that works well with the vast majority of MOF materials. For QUF-001, heating at 90 °C for 16 h was found to be the perfect temperature versus time combination. The structural integrity of the MOF lattice during such activation was confirmed by XRPD as given in Figure 3a, with complete retention of the crystallinity. At higher temperatures, the framework of QUF-001 tended to collapse. TGA analysis as provided in Figure 3b shows clear benefits of the activation process: about 4% of initial weight loss was observed in the as-synthesized Zn-MOF below 120 °C, while the material tested for thermal stability after activation showed very high thermal resistance with less than 1% (~1%) weight loss up to 275 °C. It is noteworthy that the crystal structure of QUF-001 contained also a molecule of DFM with a boiling point of 156 °C, and this was about 19% of the total weight. However, TGA analysis showed stability up to 300 °C with weight loss of 4% corresponding to water released at around 100 °C. This assumed that the coordinated DMF molecule was not released and was strongly coordinated in the Zn coordination sphere and released from samples only after 300 °C, with the peak from derivate TA at 330°C as shown in Figure 1d. A further peak from DTA at 420 °C can be attributed to thermal degradation of the TPDCA segment in QUF-001.

**Figure 4:**
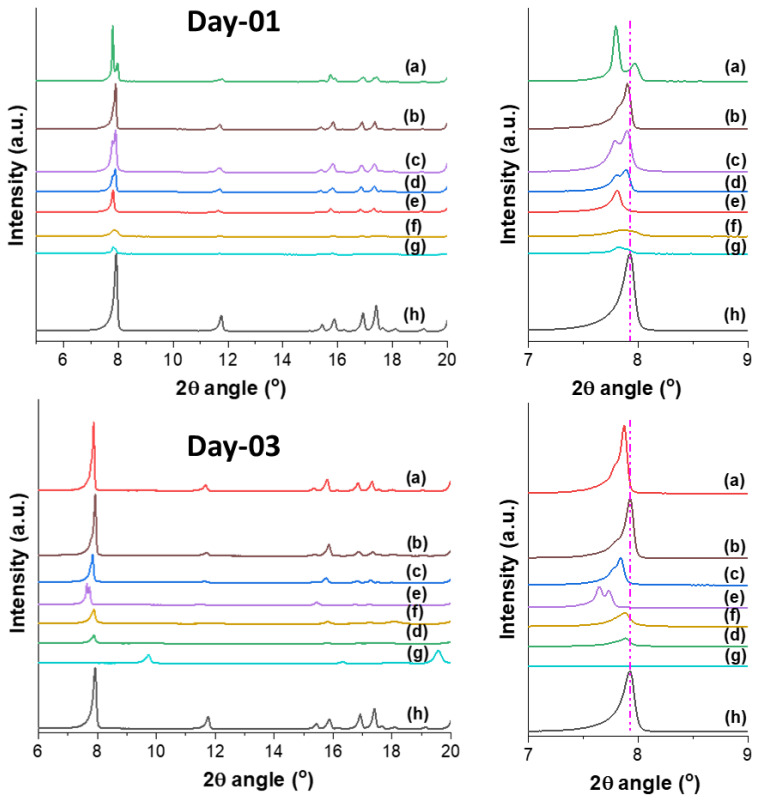
XRPD patterns taken from the QUF-001 mixed with different solvents after Day 1 (upper frames) and Day 3 (bottom frames). The solvents are: (**a**) acetonitrile, (**b**) THF, (**c**) benzene, (**d**) DCM, (**e**) hexadecane, (**f**) methanol, (**g**) isopropanol; patterns (**h**) belong to pure (no solvent) activated QUF-001. Frames on the right-hand side show enlarged view of the (001) low 2*θ* angle peak(s).

Inclusion of different organic solvents into the activated sample of QUF-001 was examined. In the testing process, activated QUF-001 particles with 10–12 nm average particle size distribution (calculated based on Scherrer equation) were kept mechanically agitated for a certain time in the respective solvent; the progress of the inclusion was followed by performing XRPD measurements after Day 1 and Day 3, with the data summarized in Figure 4.

After Day 1, QUF-001 mixed with the solvents acetonitrile, tetrahydrofuran (THF), benzene and dichloromethane (DCM) showed peak splitting around 2*θ* = 7.92°, as can be seen in Figure 4, curves a, b, c, and d. For hexadecane, no peak splitting was observed at low angle, indicating the presence of a single phase (Figure 4, curve e). Furthermore, since the (001) peak was strongly shifted to lower angle with 2*θ* = 7.78°, the obtained phase was probably a pure intercalated one. With methanol and isopropanol, a broadening of the (001) peak indicated a loss in crystallinity (Figure 4, curves f and g).

After Day 3, QUF-001 mixed with the solvents acetonitrile, THF, benzene and hexadecane still showed peak splitting around 2*θ* = 7.92°, as can be seen in Figure 4, curves a, b, c and e. This peak splitting was most probably due to the coexistence of the initial and the intercalated phase. Furthermore, the positions of the new peak (below 2*θ* = 7.92°) were at a lower angle compared to the (001) peak of QUF-001. This indicated an enlargement of the inter-reticular distance *d*_001_ and the *c* cell parameter, which was most probably due to the inclusion of the solvent in the QUF-001 structure. It should be mentioned that this process is reversible and intercalated solvent can penetrate and introduce the other phase formation. Similarly, such solvent stimulus response studies on MOF were performed previously on Zn-, Cd- and other metal-based MOFs [59,60,61]. With DCM and methanol, a broadening of the peak (001) was observed due to the loss in crystallinity (Figure 4, curves d and f). With isopropanol, a significant change in the pattern was observed compared to the other samples, and the (001) peak disappeared. This could be due either to the decomposition of the QUF-001 phase and formation of the ligand TPDCA (Figure S3), or to a structural change.

**Figure 5:**
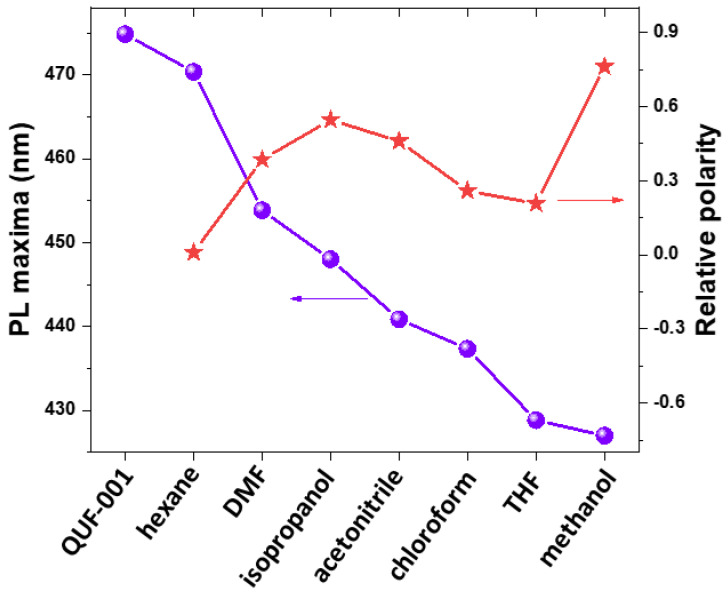
Trend in the PL maxima peak positions for activated QUF-001 and QUF-001 mixed with different solvents (violet cycles). All samples excited at 350 nm and compared with the solvent polarity (red stars).

As the next step, luminescence properties of QUF-001 were examined after incorporation of the solvents. TPDCA ligand itself exhibited a photoluminescence (PL) emission peak at 450 nm while as-prepared QUF-001 has a peak at 475 nm upon excitation at 350 nm (Figure S4). It is apparent from Figure 5 that after mixing QUF-001 with different solvents, alteration in the position of peak maxima appeared.

The effect of the used solvent on PL spectra was observed for QUF-001 and showed PL emission maxima of methanol located at 426 nm, followed further in order by THF (429.7 nm), chloroform (436 nm), acetonitrile (441 nm), isopropanol (446 nm), DMF (454 nm) and hexane (470 nm) (Figure 5).

Hexane was the most non-polar solvent employed in the present study and had PL maxima at 470 nm. Alternatively, methanol was the most polar and had PL maxima located at 426 nm; PL maxima of QUF-001 with other solvents were in between, but not in a directional order. Hence, it is reasonable to assume PL emission maxima depend on the collective effects of solvent polarity, hydrogen bonding, size of the solvent molecule and the interaction of hetero atoms in the solvent molecule with the hetero atoms in the QUF-001 MOF structure. This can be ascribed to a solvatochromic effect; however, it should be pointed out that XRD indicated changes that could have resulted from new phase formation. Thus, the changes in luminescence properties do not need to be the result of solvatochromism of QUF-001 but may also be due to other phases with different optical properties. Nevertheless, this approach can be potentially employed for sensing materials, since it gives distinct spectral lines for the individual solvent media they are in that are not overlapping.

We then studied the methane storage and CO_2_ capture capacity of QUF-001. Pre-programmed gas sorption–desorption measurements made from 0–50 bar pressure for adsorption and reversing back to zero for desorption resulted in a cumulative 20 data points for a complete cycle. According to N_2_ gas adsorption–desorption measurements shown in Figure S5, QUF-001 exhibited slight hysteresis in low and high vapor pressure regions, with a Langmuir surface area of 2.85 m^2^/g. We noticed that the sample had a BET surface area of 1.9078 m²/g, which was relatively low. It also had a BJH desorption pore diameter of ~40 nm and t-plot pore volume of 0.000610 cm³/g. The measured surface area was low for a porous material with open networks of MOF type. As can be seen from the SEM images of the MOF (Figure 2a), solvent inclusion and gas storage behavior may arise from the lamellar channels in the structure.

**Figure 6:**
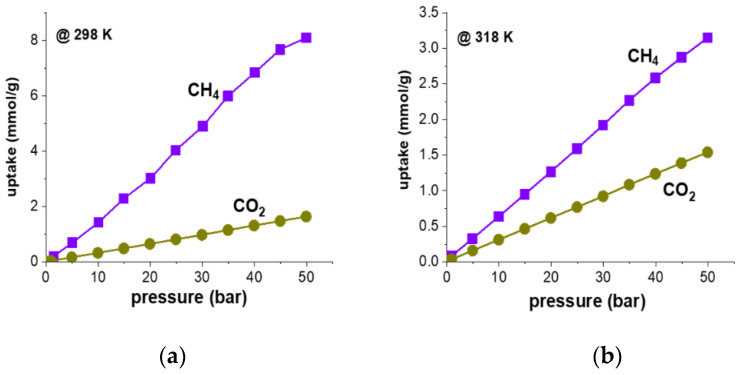
Plots of the CO_2_ (violet squares) and CH_4_ gas (green cycles) adsorption behavior of QUF-001and the uptake of these two gases as a function of the pressure, at two different temperatures of 298 K (**a**) and 318 K (**b**).

We then investigated the sorption capacity of CO_2_ and CH_4_ gases onto QUF-001 as depicted in Figure 6. High-pressure experiments were performed at 298 and 318 K isotherms. A complete adsorption–desorption cycle passed through stepwise pressure increases and decreases with each adsorbate was carried out from vacuum to 50 bars and back to vacuum at the end of the measurements to observe the hysteresis behavior. At each isotherm, there were a total of 12 adsorption and 8 desorption data points collected for QUF-001, which are presented in Figures S6–S9. At first glance, all of those curves demonstrated a smooth increasing trend with increasing pressure. However, adsorption and desorption data points showed almost the same values, which showed that there was no hysteresis and no significant changes occurred in the samples during the overall pressure loop. We noticed that in order to obtain reliable data, peripheral conditions such as humidity, ambient pressure, and temperature had to be considered [7]. To prevent potential irreversible structure collapse and reduction of the surface area as well as pore volume due to moisture within the measurement chamber, samples were degassed. Furthermore, a Drierite column was used to pre-dry the gases. A similar sorption–desorption overlapped trend of variation was observed while studying a (NH_4_)_2_Mg(H_2_P_2_O_7_)_2_•2H_2_O) single-crystal sample with CO_2_ and CH_4_ at isotherms 298 and 318 K [62]. Figures S9 and S10 confirm the expected thermodynamic trend of variation, namely that the sorptivity of both used gases decreased upon increasing the temperature and increased upon increasing the pressure. However, all of the isotherms with CO_2_ and CH_4_ with QUF-001 were completely reversible and the absence of hysteresis confirmed the advantages of reusability and cost efficiency of this MOF under primary vacuum [3,7,63]. Sorption results showed that CO_2_ (1.6413 mmol/g) sorption was significantly lower than that for CH_4_ (8.0907 mmol/g) at temperature 298 K and pressure of 50 bars, which meant that QUF-001 had higher affinity to capture CH_4_ as compared to CO_2_. Clear evidence of QUF-001 CH_4_ sorption selectivity over CO_2_ gas at each temperature and pressure showed that the use of this MOF may be beneficial for the chemical and petroleum industries in terms of CH_4_ separation from CH_4_/CO_2_ mixtures [7]. From Figure 6, at temperature 298 K, between 45 to 50 bar pressure, the sorption curve seems flattened. Typically, MOFs, covalent organic frameworks and covalent organic polymers follow type IV adsorption isotherms, showing finite multi-layer adsorption corresponding to complete filling of the capillaries and pores [64]. The adsorption isotherm profiles (Figure 6) rather fell within the type III behavior, indicating weak substrates and the formation of multilayers. Here, there was no flattish region in the curve assuming lack of a monolayer. In one of our studies, the CO_2_ sorption capacities of Rb_2_Co(H_2_P_2_O_7_)_2_•2H_2_O were higher than those of the currently investigated sample, but CH_4_ sorption efficiency was 3.5-fold better in QUF-001. Although there was no clear superiority among either CO_2_, CH_4_ or other gases for MOF structures, the trend in ranking the sorption performance of such gases showed that the CO_2_ capture performance of MOFs was higher than that for methane [65,66,67]. We also showed a similar trend through gas sorption demonstrations on MOF-5 previously [68,69]. Moreover, on comparing the CO_2_ sorption data of this work with hydroxy metal carbonates M(CO_3_)_x_(OH)_y_ (M = Zn, Zn-Mg, Mg, Mg-Cu, Cu, Ni, and Pb) [70], Rb_2_Co(H_2_P_2_O_7_)_2_•2H_2_O showed higher values at 35 bar and 318 K, although the hydroxy metal carbonates were measured at 316 K.

## 3. Materials and Methods

### 3.1. Chemicals

Citric acid, L-cysteine, *N,N*-dimethylformamide (DMF), Zn(NO_3_)_2_•6H_2_O, Cu(NO_3_)_2_•3H_2_O, Fe(NO_3_)_3_•9H_2_O, 37% hydrochloric acid, absolute ethanol, acetone, acetonitrile, benzene, methanol, tetrahydrofuran (THF), dichloromethane (DCM), hexadecane (HD), propanol, chloroform and isopropanol were purchased at the highest purity level available from Sigma Aldrich and used as received without further purification. Deionized water was obtained from Millipore system.

### 3.2. Preparation of MOFs

(Step 1) Synthesis of the ligand, TPDCA.

TPDCA ligand was prepared according to the procedure published previously [39] (Figure S1). A 28.7 g amount of anhydrous citric acid was mixed with 18.1 g of l-cysteine in a flat-bottom flask and autoclaved at 150 °C for 4 h. The reaction product was separated and recrystallized in acetone. The structure of the TPDCA was confirmed by ^1^H NMR (Figure S10) and ^13^C NMR (Figure S11) prior to employing it in the subsequent MOF preparation.

^1^H NMR (δ,d_6_ DMSO, 600 MHz) ppm: 13.58 (broad singlet, 2H, -COO-H), 6.56 (doublet, *J* = 1.5 Hz, 1H, Ar-*H*), 6.51 (doublet, *J* = 1.5 Hz, 1H, Ar-*H*), 5.43 (doublet- doublet, *J* = 1.5 & 8.5 Hz, 1H, HOOC-C-*H*), 3.87 (doublet-doublet, *J* = 8.5 & 11.6 Hz, 1H, H-C-*H*), 3.57 (doublet-doublet, *J* = 1.5 & 11.6 Hz, 1H, H-C-*H*).

^13^C NMR ^1^H NMR (δ,d_6_ DMSO, 150 MHz) ppm: 169.2, 165.6, 160.6, 149.9, 142.7, 115.0, 97.94, 62.6, 31.6.

(Step 2) Synthesis of the MOFs: In a typical synthesis process, 50.0 mg of the TPDCA ligand and 61.72 mg of Zn(NO_3_)_2_.6H_2_O for QUF-001, 50.01 mg of Cu(NO_3_)_2_ for QUF-002 and 83.63 mg of Fe(NO_3_)_3_ for QUF-003 were placed into a dry flask. Then, 10 mL of DMF was added and acidified with a drop of concentrated HCl acid. The solution was incubated in an oven at 80 °C for 96 h under Ar atmosphere. The solids formed were separated by centrifugation and dried under vacuum at 60 °C for 16 h. Reaction yield was 72, 52 and 45% for QUF-001, QUF-002 and QUF-003, respectively.

### 3.3. Characterization

X-ray powder diffraction (XRPD) patterns of the samples were recorded on a PANalytical empyrean machine equipped with Cu Kα radiation as the X-ray source [71]. Measurements were made between 5 and 40° 2*θ* angles. Fourier transform infra-red (FTIR) measurements were performed on a PerkinElmer Frontier device with ZnSe ATR unit in the wavenumber region from 4000–500 cm^−1^ in the transmittance mode at a scan rate of 64 scans per cycle [72]. Thermal stability of the samples was assessed using thermogravimetric analysis (TGA) employing 10 mg sample on a TGA 4000 device by PerkinElmer under N_2_ atmosphere [73]. Scanning electron microscopy (SEM) imaging was performed on a ZEISS SIGMA 500 VP FE SEM device under different magnifications [74].

### 3.4. Single Crystal X-ray Diffraction Measurements

Single crystals of QUF-001 suitable for X-ray diffraction were selected on the basis of the size and sharpness of their diffraction spots. Data collection was carried out on a D8 venture diffractometer using Mo*Kα* radiation. Data processing and all refinements were performed with the Jana2006 program package [75]. A multi-scan-type absorption correction was applied using SADABS [76], and the crystal shape was determined using a microscope.

### 3.5. Solvent Inclusion

Two milligrams of activated QUF-001 were placed in a glass vial filled with 2 mL of the respective solvent. Solvents used were acetonitrile, benzene, methanol, THF, DCM, hexadecane, and isopropanol. The contents in the vials were aged for 1 day and 3 days. For XRD measurements, solid powder was separated from the solvent by decanting, and the solid was spread on a Petri dish to facilitate air drying for 2 h; dried powders were collected and characterized.

### 3.6. Gas Sorption Measurements

Ten milligrams of QUF-001 were dried overnight at 90 °C for activation of the MOF material and stored in a desiccator connected to vacuum until the crystals were used. Gas adsorption–desorption tests were performed for pure CO_2_ and CH_4_ gases and the mixture of CO_2_ and CH_4_ on a Rubotherm magnetic suspension sorption apparatus (MSA), which operated on the basis of Archimedes’ buoyancy principle. Pressure in the sample bucket was increased from vacuum to a predefined high-pressure value (0–50 bar) in the adsorption process, and was reversed for the desorption. At the very beginning of the test, the sample bucket was held at vacuum for 10 h to ensure complete surface degassing. Gas adsorption–desorption measurements were performed at two different temperatures, 298 K and 318 K. Buoyancy correction was carried out for the sorption measurements as well. The details of the correction process were explained in detail previously [6].

Pressure transducers (Paroscientific, Redmond, WA, USA) worked from vacuum up to 350 bar with an uncertainty of 0.01% of the full scale (*u(P)* 0.035 bar), whereas the temperature sensor (Minco PRT, Fridley, MN, USA) had a measurement accuracy of 0.5 K (u(T) = 0.05 K).

## 4. Conclusions

In summary, we successfully synthesized fluorescent and thermally stable Zn(II)-based MOF as a probe for solvent and gas adsorption. Fluorescent organic ligand TPDCA obtained from easily accessible, sustainable precursors citric acid and cysteine was applied for the first time for MOF structure fabrication. Single crystal analysis of the QUF-001 sample confirmed the two-dimensional lamellar structure of this MOF, where TPDCA ligand coordinated to Zn(II) central atoms, along with the incorporation of DMF. Solvent molecules inclusion led to tunability of the diffraction pattern as well as shifts in emission maxima, which indicated the solvent inclusion within the interlamellar space in the two-dimensional QUF-001 structure. Gas sorption properties of QUF-001 for CO_2_ and CH_4_ were examined and determined at 1.6 mmol/g and 8.1 mmol/g, respectively, at temperature 298 K and the pressure of 50 bars. Thus, our study offers a platform for the application of cheap and accessible ligand TPDCA for fluorescent MOFs with tailored properties.

## Figures and Tables

**Table 1:** Crystal data and structure refinement of QUF-001.

**Crystal Data**	
Chemical formula	C_12_H_12_N_2_O_6_SZn × 0.25H_2_O
*M*_r_	382.2
Crystal system, space group	Triclinic, *P*-1
Temperature (K)	293
*a*, *b*, *c* (Å)	6.4459 (3), 10.4427 (5), 11.2947 (5)
α, β, γ (°)	88.693 (2), 83.751 (3), 77.741 (2)
*V* (Å^3^)	738.52 (6)
*Z*	2
Radiation type	Mo *K*α
µ (mm^−1^)	1.84
Crystal size (mm)	0.06 × 0.03 × 0.01
**Refinement**	
R[*F*^2^ > 2σ(*F*^2^)], *wR*(*F*^2^), *S*	0.042, 0.106, 1.09
No. of reflections	3269
No. of parameters	203
No. of restraints	0
H-atom treatment	H-atom parameters constrained
Δρ_max_, Δρ_min_ (e Å^−3^)	0.93, −0.62

## Data Availability

Not applicable.

## Supplementary Materials

The following supporting information can be downloaded at: https://www.mdpi.com/article/10.3390/molecules27123845/s1, Figure S1. Synthesis of TPDCA and related MOF. Figure S2. Full pattern matching of the XRPD pattern of the QUF-001 compound. Figure S3. XRPD patterns of (a) TPDCA, (b) QUF-001 with the solvent isopropanol and (c) pure QUF-001. Figure S4. Emission spectra of TPDCA (red line) and QUF-001 (black line), excited at 375 nm. Figure S5. N_2_ gas adsorption–desorption measurements of QUF-001. Figure S6: CO_2_ absorption–desorption plot at 25 °C for QUF-001. Figure S7: CO_2_ absorption–desorption plot at 45 °C for QUF-001.Figure S8: CH_4_ absorption–desorption plot at 25 °C for QUF-001. Figure S9: CH_4_ absorption–desorption plot at 45 °C for QUF-001.Figure S10. ^1^H spectrum of TPDCA. Figure S11. ^13^C NMR spectra of TPDCA. Table S1. Structure refinement for QUF-001. Table S2. Fractional atomic coordinates and isotopic atomic displacement parameters (Å2) for C12H12N2O6SZn × 0.25H2O. Table S3. Anisotropic displacement parameters (Å2) for QUF-001. The anisotropic displacement factor exponent takes the form: −2π^2^[(*ha**)2*U*_11_+...+2*hka***b***U*_12_].
