# Supplementary material for: Fluorescent Zn(II)-Based Metal-Organic Framework: Interaction with Organic Solvents and CO2 and Methane Capture
Source: Molecules. 2022 Jun 15;27(12):3845. doi: 10.3390/molecules27123845 (PMC9228242; doi:10.3390/molecules27123845)
Supplement: Supplementary file 1 [file molecules-27-03845-s001.zip › molecules-1741971-supplementary.pdf]

molecules

Supplementary Materials

Fluorescent Zn(II)-Based Metal-Organic Framework: Interaction with Organic Solvents and CO_2 and Methane Capture

Sifani Zavahir [1], Hamdi Ben Yahia [2], Julian Schneider [3], DongSuk Han [1], Igor Krupa [1], Tausif Altamash [2], Mert Atilhan [4], Abdulkarem Amhamed [2,*] and Peter Kasak [1,*]

[1] Center for Advanced Materials, Qatar University, Doha P.O. Box 2713, Qatar; fathima.z@qu.edu.qa (S.Z.); dhan@qu.edu.qa (D.H.); igor.krupa@qu.edu.qa (I.K.)
[2] Qatar Environment & Energy Research Institute, Hamad Bin Khalifa University, Doha 34110, Qatar; benyahia_hamdi@yahoo.fr (H.B.Y.); taltamash@hbku.edu.qa (T.A.); aamhamed@hbku.edu.qa (A.A.)
[3] Department of Materials Science and Engineering, and Center for Functional Photonics (CFP), City University of Hong Kong, 83 Tat Chee Avenue, Hong Kong 999077, China; julianschneider86@gmx.de
[4] Department of Chemical and Paper Engineering, Western Michigan University, Kalamazoo, MI 49008, USA; mert.atilhan@wmich.edu
* Correspondence: aamhamed@hbku.edu.qa (A.A.); peter.kasak@qu.edu.qa (P.K.)

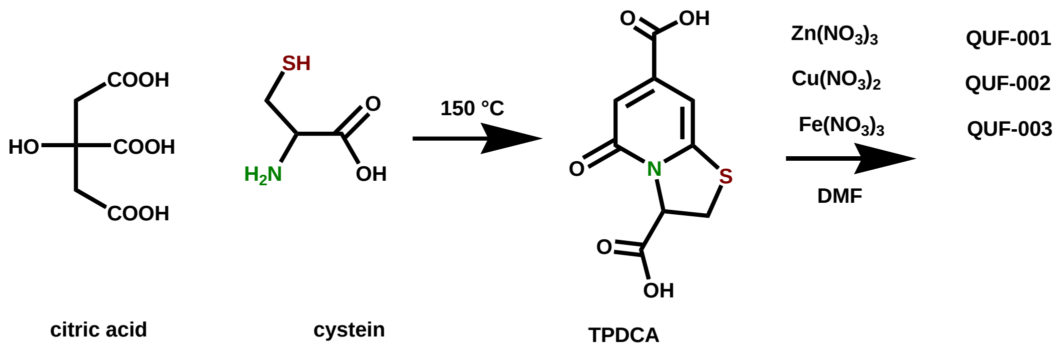

Figure S1. Synthesis of TPDCA and related MOF.

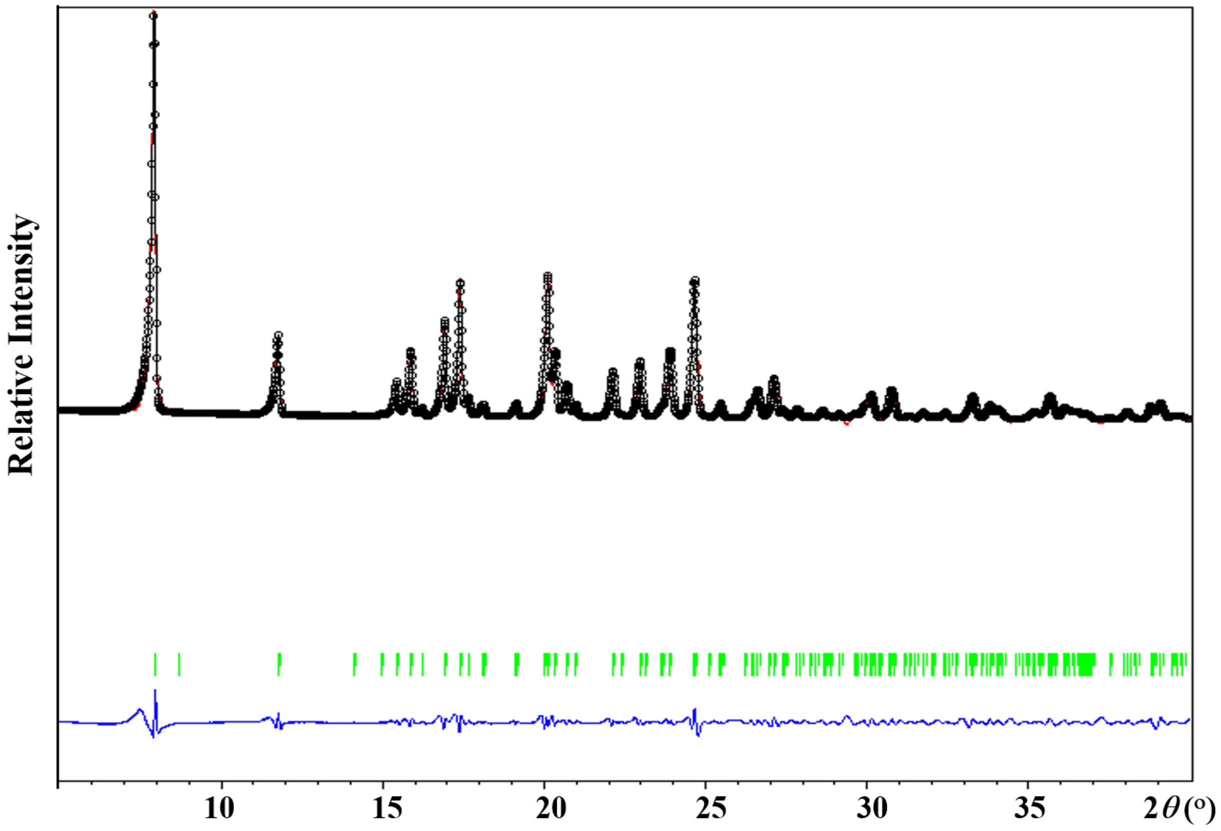

Figure S2. Full pattern matching of the XRPD pattern of the QUF-001 compound.

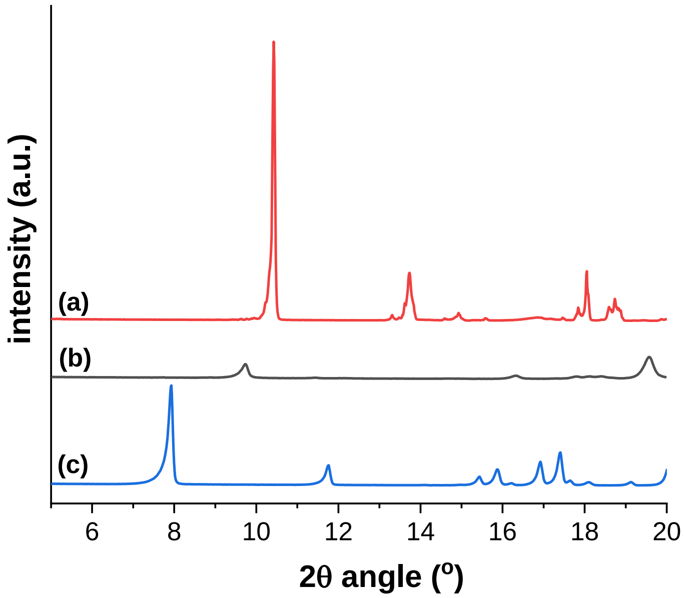

Figure S3. XRPD patterns of (a) TPDCA, (b) QUF-001 with a solvent isopropanol and (c) pure QUF-001.

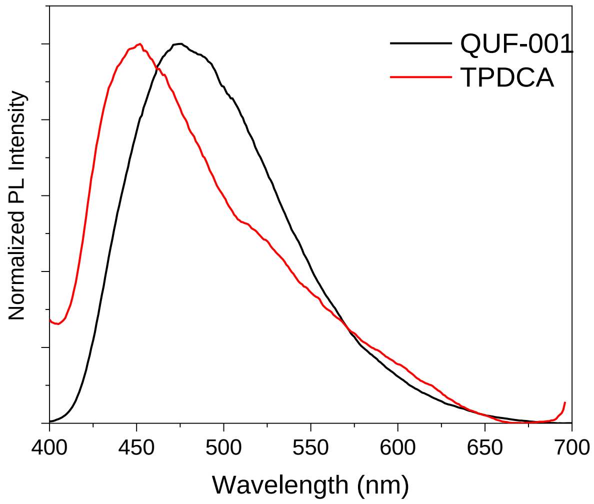

Figure S4. Emission spectra of TPDCA (red line) and QUF-001 (black line), excited at 375 nm.

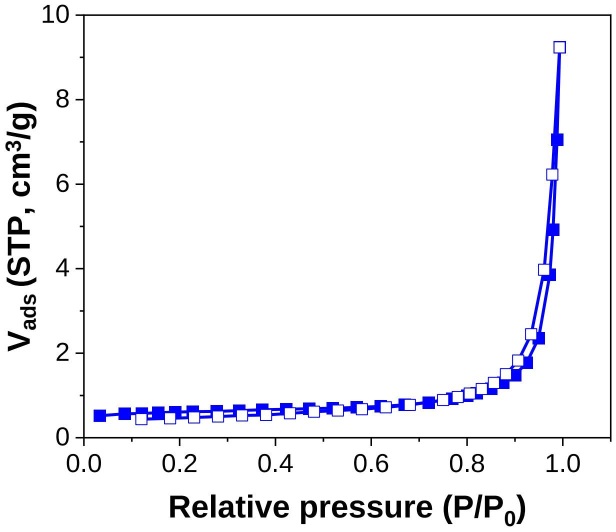

Figure S5. N_2 gas adsorption-desorption measurements of QUF-001.

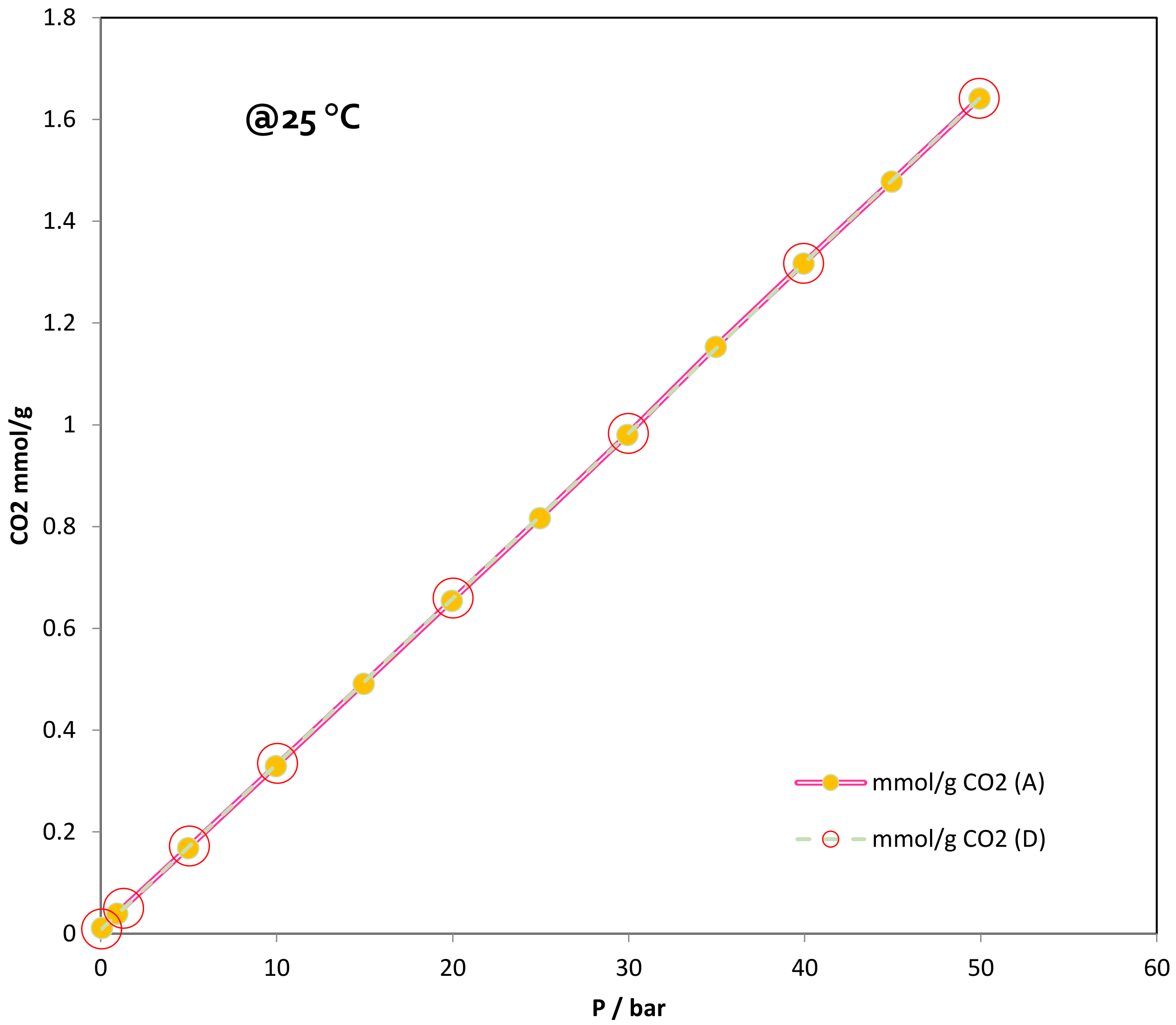

Figure S6. CO_2 absorption-desorption plot at 25 °C for QUF-001.

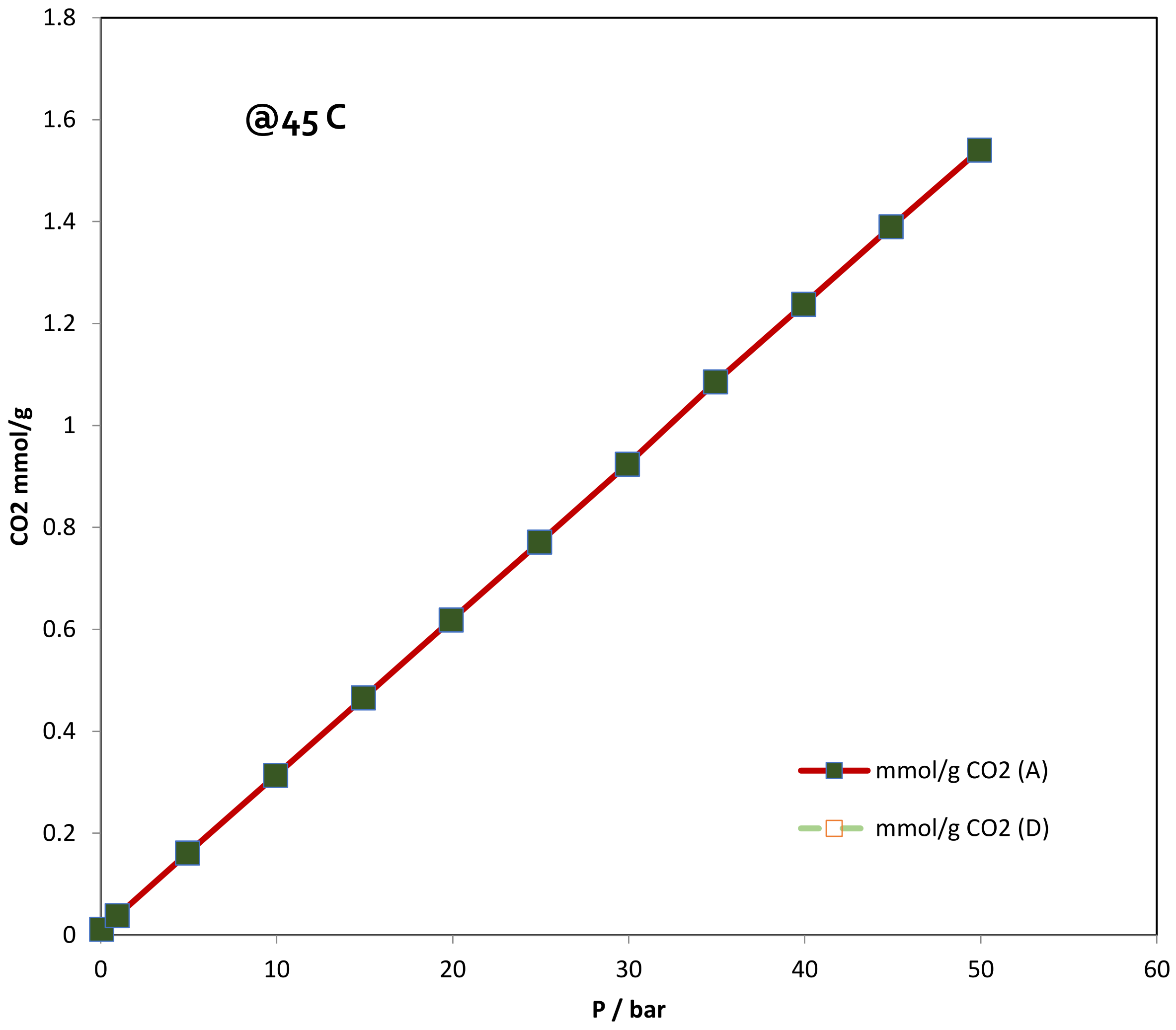

Figure S7. CO_2 absorption-desorption plot at 45 °C for QUF-001.

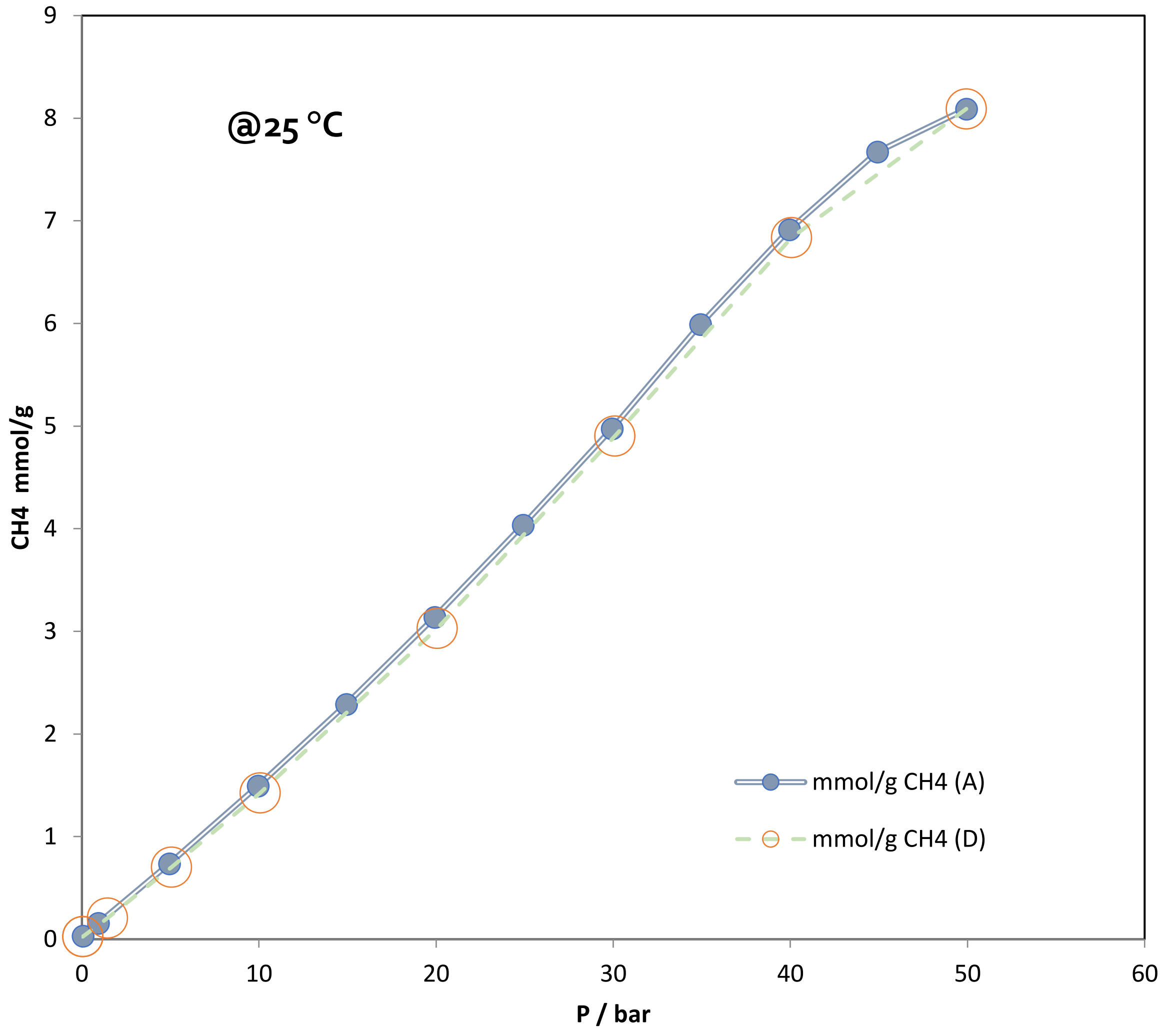

Figure S8. CH_4 absorption-desorption plot at 25 °C for QUF-001.

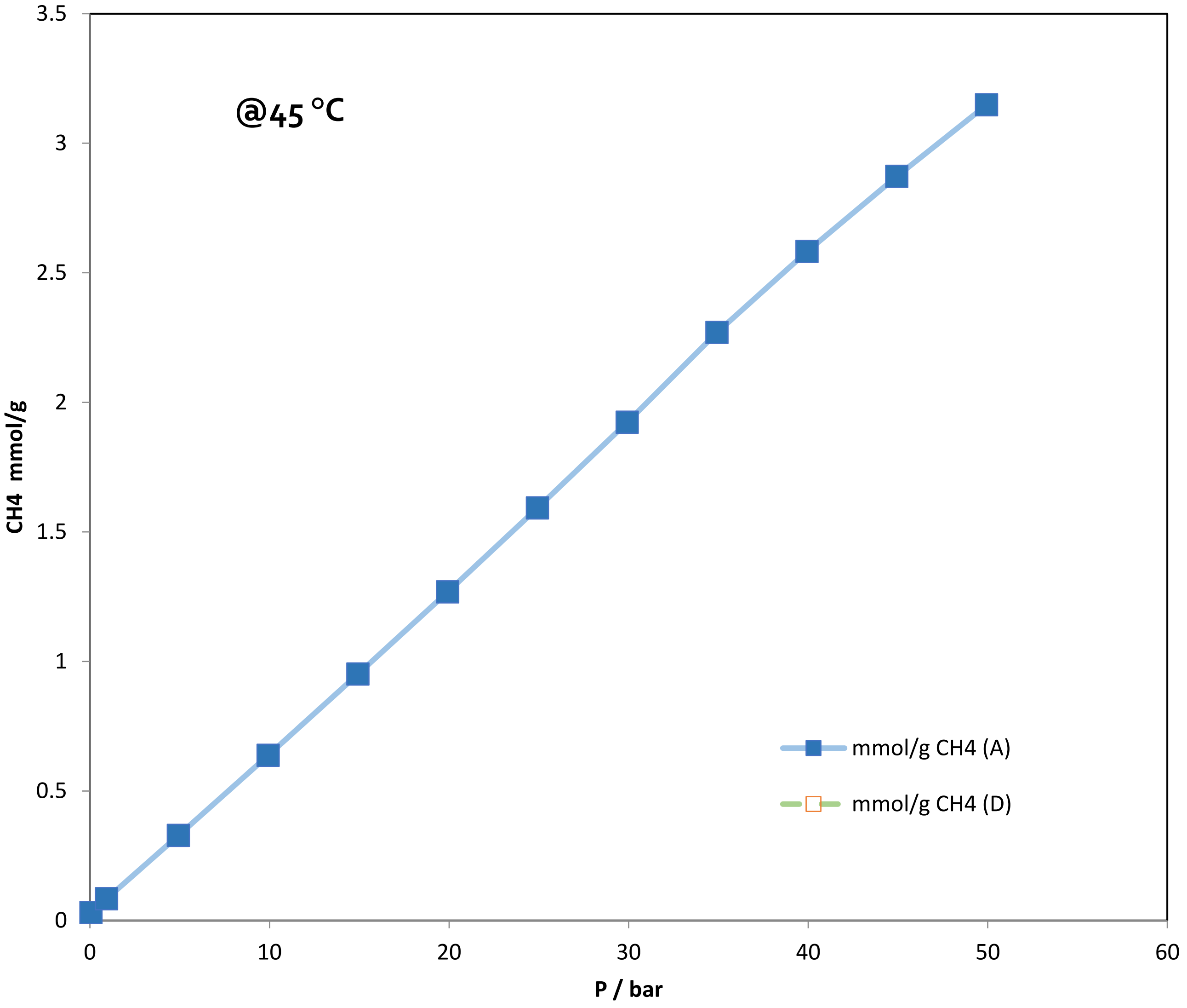

Figure S9. CH_4 absorption-desorption plot at 45 °C for QUF-001.

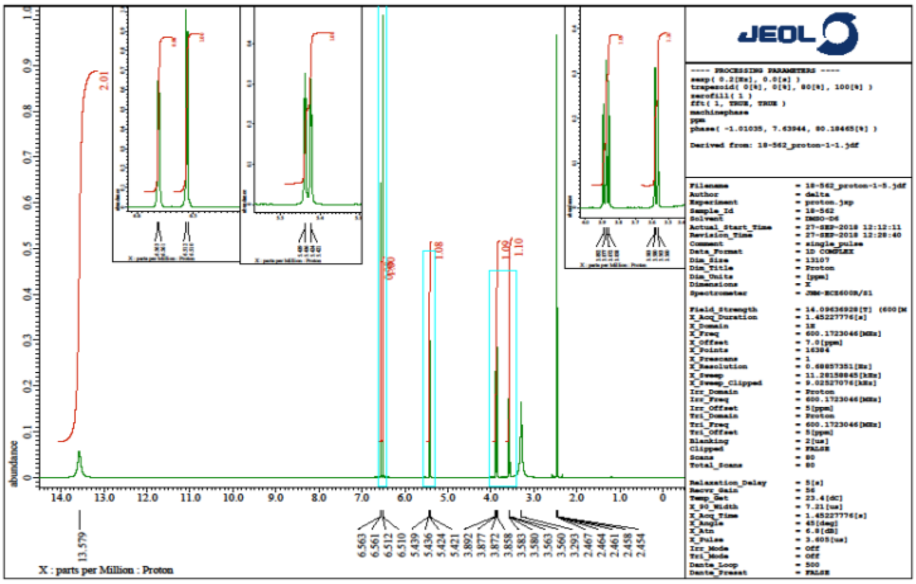

Figure S10. ^{1}H spectrum of TPDCA.

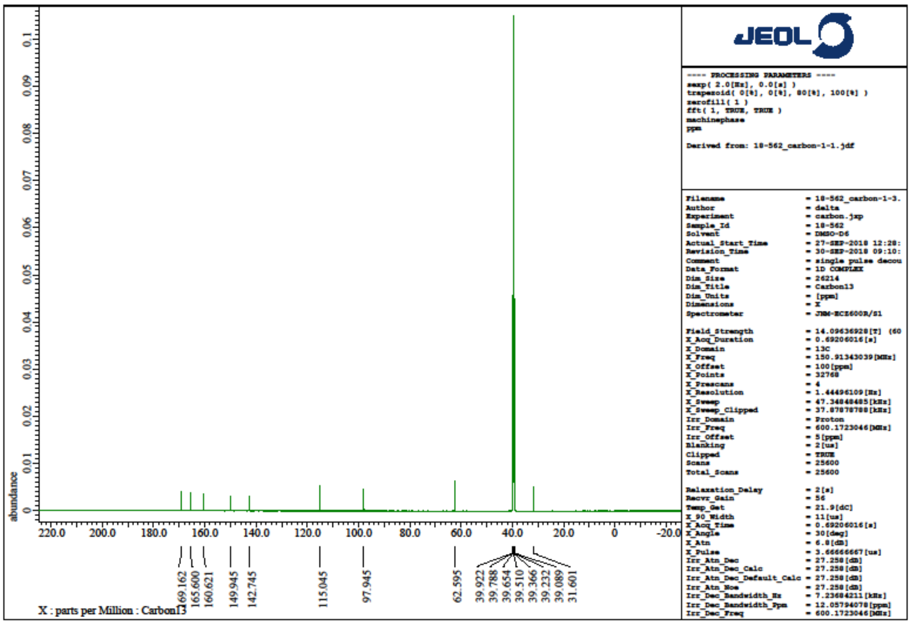

Figure S11. ^{13}C NMR spectra of TPDCA.

Table S1. Structure refinement for QUF-001.

Data collection	
**Diffractometer**	D8 venture diffractometer
Absorption correction	Multi-scan *SADABS*
T_{min}, T_{max}	0.87, 0.89
No. of measured, independent and observed [$I > 3\sigma(I)$] reflections	27964, 3269, 1762
R_{int}	0.151
$(\sin \theta/\lambda)_{max}$ (Å^{-1})	0.641

Refinement	
$R[F^2 > 2\sigma(F^2)]$, $wR(F^2)$, S	0.042, 0.106, 1.09
No. of reflections	3269
No. of parameters	203
No. of restraints	0
H-atom treatment	H-atom parameters constrained
$\Delta\rho_{max}$, $\Delta\rho_{min}$ (e Å^{-3})	0.93, −0.62

Table S2. Fractional atomic coordinates and isotopic atomic displacement parameters (Å^2) for $C_{12}H_{12}N_2O_6SZn \times 0.25H_2O$.

Atom	*Wyck.*	*Occ.*	*x*	*y*	*z*	*Ueq (Å^2)*
Zn1	2*i*	1	0.80765(8)	0.27583(5)	0.32628(5)	0.03196(18)
O1	2*i*	1	0.8870(4)	0.4378(3)	0.3639(3)	0.0460(12)
O2	2*i*	1	0.5837(5)	0.5613(3)	0.3135(3)	0.0531(13)
O3	2*i*	1	1.0327(5)	0.1350(3)	0.3705(3)	0.0450(12)
O4	2*i*	1	0.5524(4)	0.2623(2)	0.4377(2)	0.0349(10)
O5	2*i*	1	0.6989(4)	0.2718(3)	0.1718(3)	0.0452(12)
O6	2*i*	1	1.2263(6)	0.1535(4)	0.2023(4)	0.0814(17)
C1	2*i*	1	0.7515(6)	0.5445(4)	0.3581(4)	0.0353(16)
C2	2*i*	1	0.8187(6)	0.6598(3)	0.4173(4)	0.0325(15)
C3	2*i*	1	1.0312(6)	0.6848(4)	0.3600(4)	0.0438(18)
C4	2*i*	1	0.2894(6)	0.1454(4)	0.6955(4)	0.0311(15)
C5	2*i*	1	0.4334(6)	0.0407(4)	0.7300(4)	0.0346(15)
C6	2*i*	1	0.6343(6)	0.0087(4)	0.6624(4)	0.0298(15)
C7	2*i*	1	1.1959(7)	0.1030(4)	0.2993(4)	0.0364(17)
C8	2*i*	1	0.6770(6)	0.0784(3)	0.5624(4)	0.0289(14)
C9	2*i*	1	0.5273(6)	0.1887(4)	0.5272(4)	0.0287(15)
C10	2*i*	1	0.5185(7)	0.3354(4)	0.1533(4)	0.0464(19)
C11	2*i*	1	0.5488(11)	0.2564(7)	-0.0437(5)	0.113(4)
C12	2*i*	1	0.2209(8)	0.4055(6)	0.0408(5)	0.078(3)
N1	2*i*	1	0.3372(5)	0.2179(3)	0.5976(3)	0.0275(12)
N2	2*i*	1	0.4389(6)	0.3377(4)	0.0528(4)	0.0514(16)
S1	2*i*	1	0.03459(18)	0.20847(12)	0.76504(12)	0.0503(5)
H1c2	2*i*	1	0.82808	0.63550	0.49934	0.0390*
H1c3	2*i*	1	1.12098	0.60351	0.33159	0.0525*
H2c3	2*i*	1	1.09384	0.72913	0.41572	0.0525*
H1c5	2*i*	1	0.39947	-0.01006	0.79847	0.0415*
H1c8	2*i*	1	0.81172	0.05220	0.51485	0.0346*
H1c10	2*i*	1	0.43314	0.38606	0.21790	0.0557*
H1c11	2*i*	1	0.52964	0.30304	-0.11732	0.1361*
H2c11	2*i*	1	0.69815	0.23306	-0.03382	0.1361*
H3c11	2*i*	1	0.49259	0.17845	-0.04501	0.1361*
H1c12	2*i*	1	0.21526	0.44940	-0.03475	0.0941*
H2c12	2*i*	1	0.13006	0.34322	0.04553	0.0941*
H3c12	2*i*	1	0.17326	0.46861	0.10369	0.0941*

O7w	2*i*	0.25	0.901(3)	-0.031(3)	0.0102(18)	0.147(8) *
H1o7w	2*i*	0.25	0.80629	-0.01140	-0.03374	0.1765*
H2o7w	2*i*	0.25	0.89117	-0.08237	0.06506	0.1765*

*: $U_{iso.}$

Table S3. Anisotropic displacement parameters (Å^2) for QUF-001. The anisotropic displacement factor exponent takes the form: $-2\pi^2[(ha^*)^2U_{11}+...+2hka^*b^*U_{12}]$.

Atom	U_{11}	U_{22}	U_{33}	U_{12}	U_{13}	U_{23}
Zn1	0.0325(3)	0.0242(3)	0.0371(3)	0.00122(20)	-0.0092(2)	0.0019(2)
O1	0.0385(17)	0.0194(15)	0.078(2)	0.0052(13)	-0.0177(16)	-0.0039(15)
O2	0.044(2)	0.0348(18)	0.082(3)	-0.0006(15)	-0.0304(19)	-0.0101(16)
O3	0.0338(18)	0.0349(17)	0.056(2)	0.0133(14)	0.0001(16)	0.0107(15)
O4	0.0305(16)	0.0284(15)	0.0402(19)	0.0042(12)	-0.0022(14)	0.0088(14)
O5	0.0350(18)	0.054(2)	0.044(2)	0.0005(15)	-0.0119(15)	-0.0022(16)
O6	0.055(2)	0.087(3)	0.084(3)	0.015(2)	0.006(2)	0.049(2)
C1	0.031(2)	0.027(2)	0.047(3)	-0.0016(19)	-0.010(2)	0.003(2)
C2	0.024(2)	0.025(2)	0.045(3)	0.0029(17)	-0.009(2)	-0.0006(19)
C3	0.030(2)	0.035(3)	0.067(4)	-0.003(2)	-0.012(2)	-0.004(2)
C4	0.024(2)	0.031(2)	0.037(3)	-0.0040(18)	0.0001(19)	-0.003(2)
C5	0.034(2)	0.028(2)	0.038(3)	-0.0010(19)	-0.004(2)	0.0035(19)
C6	0.028(2)	0.024(2)	0.038(3)	-0.0041(17)	-0.006(2)	-0.0021(19)
C7	0.033(3)	0.025(2)	0.052(3)	-0.0074(19)	-0.008(2)	0.011(2)
C8	0.023(2)	0.022(2)	0.039(3)	0.0024(17)	-0.0035(19)	-0.0065(19)
C9	0.026(2)	0.024(2)	0.036(3)	-0.0033(18)	-0.005(2)	-0.0044(19)
C10	0.044(3)	0.049(3)	0.046(3)	-0.007(2)	-0.009(3)	0.002(2)
C11	0.085(5)	0.196(8)	0.044(4)	0.009(5)	-0.011(4)	-0.035(5)
C12	0.057(4)	0.090(5)	0.088(5)	-0.005(3)	-0.030(3)	0.026(4)
N1	0.0252(18)	0.0197(17)	0.037(2)	0.0002(14)	-0.0082(16)	-0.0014(15)
N2	0.049(3)	0.066(3)	0.040(3)	-0.008(2)	-0.019(2)	0.012(2)
S1	0.0339(7)	0.0516(8)	0.0580(9)	0.0004(6)	0.0082(6)	0.0039(6)
